# Effects of IMU sensor-to-segment calibration on clinical 3D elbow joint angles estimation

**DOI:** 10.3389/fbioe.2024.1385750

**Published:** 2024-05-17

**Authors:** Alessandro Bonfiglio, David Tacconi, Raoul M. Bongers, Elisabetta Farella

**Affiliations:** ^1^ Information Engineering and Computer Science Department (DISI), University of Trento, Trento, Italy; ^2^ Euleria Health, Rovereto, Italy; ^3^ Energy Efficient Embedded Digital Architectures, Fondazione Bruno Kessler (FBK), Trento, Italy; ^4^ Department of Human Movement Sciences, University Medical Center Groningen, Groningen, Netherlands

**Keywords:** IMU, sensor-to-segment calibration, joint angle modelling, carrying angle, elbow biomechanics

## Abstract

**Introduction:** Inertial Measurement Units (IMU) require a sensor-to-segment calibration procedure in order to compute anatomically accurate joint angles and, thereby, be employed in healthcare and rehabilitation. Research literature proposes several algorithms to address this issue. However, determining an optimal calibration procedure is challenging due to the large number of variables that affect elbow joint angle accuracy, including 3D joint axis, movement performed, complex anatomy, and notable skin artefacts. Therefore, this paper aims to compare three types of calibration techniques against an optical motion capture reference system during several movement tasks to provide recommendations on the most suitable calibration for the elbow joint.

**Methods:** Thirteen healthy subjects were instrumented with IMU sensors and optical marker clusters. Each participant performed a series of static poses and movements to calibrate the instruments and, subsequently, performed single-plane and multi-joint tasks. The metrics used to evaluate joint angle accuracy are Range of Motion (ROM) error, Root Mean Squared Error (RMSE), and offset. We performed a three-way RM ANOVA to evaluate the effect of joint axis and movement task on three calibration techniques: N-Pose (NP), Functional Calibration (FC) and Manual Alignment (MA).

**Results:** Despite small effect sizes in ROM Error, NP displayed the least precision among calibrations due to interquartile ranges as large as 24.6°. RMSE showed significant differences among calibrations and a large effect size where MA performed best (RMSE = 6.3°) and was comparable with FC (RMSE = 7.2°). Offset showed a large effect size in the calibration*axes interaction where FC and MA performed similarly.

**Conclusion:** Therefore, we recommend MA as the preferred calibration method for the elbow joint due to its simplicity and ease of use. Alternatively, FC can be a valid option when the wearer is unable to hold a predetermined posture.

## 1 Introduction

**FIGURE 1:**
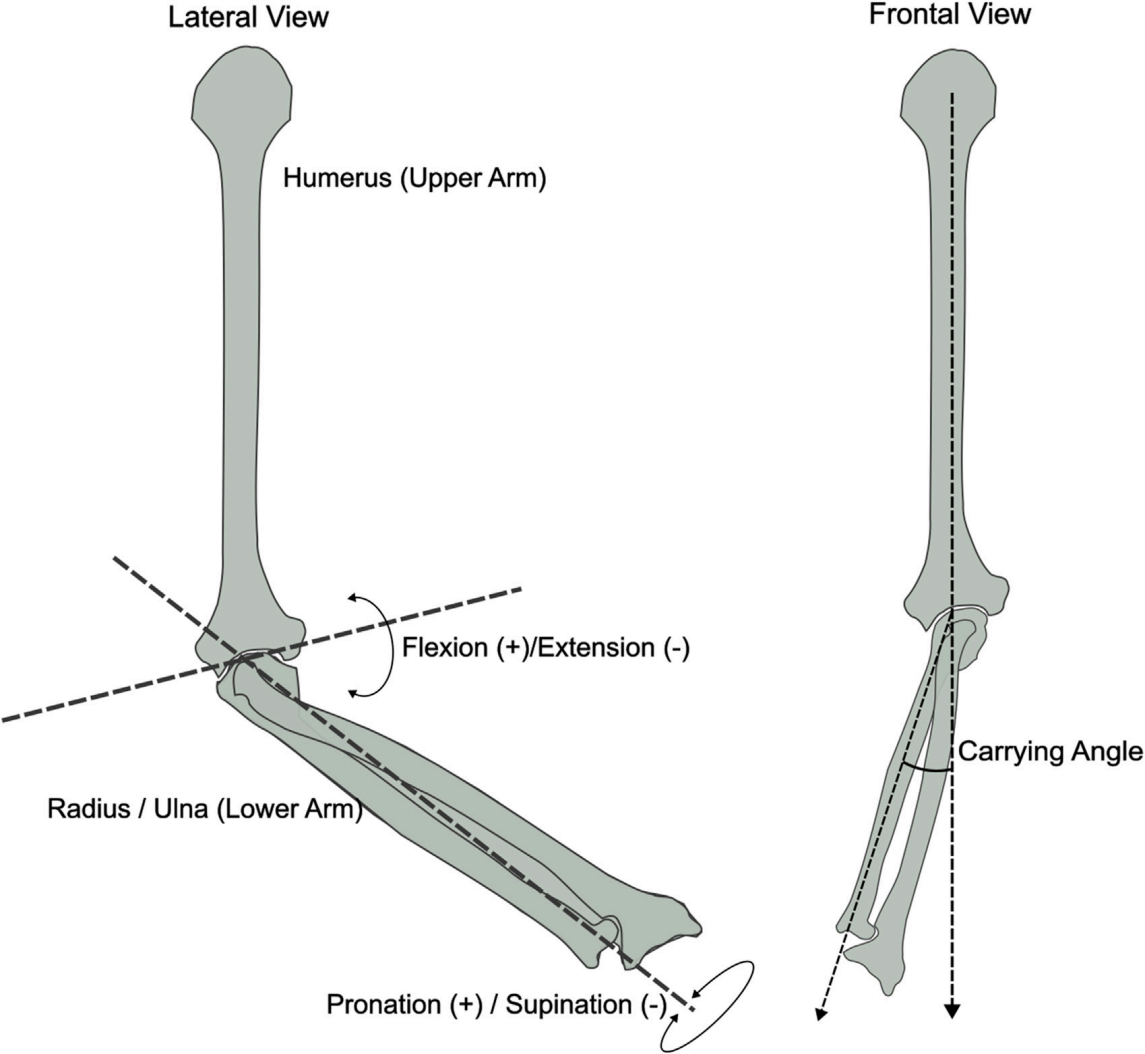
Frontal and lateral view of the elbow bone anatomy displaying the joint axes: Flexion/Extension, Pronation/Supination and Carrying Angle. Inspired by (An et al., 1983).

In clinical applications, Inertial Measurement Units (IMU) have been widely used to identify movement disorders otherwise imperceptible to the naked eye (Zadeh et al., 2023; Bo et al., 2022; Lind et al., 2023). However, despite their flexibility, low cost, and reliability, IMU require a preliminary step before they can be used to estimate the joint angle of adjacent body segments. This procedure, called sensor-to-segment calibration, involves aligning the IMU’s internal reference frame with the anatomical reference frame of the bone where the sensor is placed (Filippeschi et al., 2017; Vitali and Perkins, 2020). The elbow joint’s calibration process is particularly challenging due to its complex anatomy (Cutti et al., 2005; Cutti et al., 2006; Cutti et al., 2008). The elbow joint is anatomically composed of the humeroulnar joint, responsible for the flexion/extension movement, and the radioulnar joint, responsible for pronation/supination (Figure 1). The rotation axes associated with these two joints are approximately perpendicular to one another and the distance between the two centres of rotation is approximately 4 mm (Veeger et al., 1997). This gap generates a third angle, known as the “carrying angle” (Figure 1), which varies among subjects depending on their anatomy, age and sex (Paraskevas et al., 2004; Tükenmez and Dem, 2004), as well as being slightly dependent on elbow flexion angle (An et al., 1983).

To accurately identify the two main elbow rotation axes, scientific literature presents several techniques that are most commonly adopted to perform IMU calibration, which are more extensively described in (Fang et al., 2023). In short, these are 1) N-Pose calibration (NP), which involves holding a known pose to align each sensor reference frame to the reference of the bone underneath (Zhang and Wu, 2011; Liu et al., 2019; Humadi et al., 2021); 2) Functional calibration (FC), which consists of performing single-plane elbow flexion-extension and pronation-supination movements to estimate the relative joint rotation axis (Cutti et al., 2008; Ligorio et al., 2017); 3) Manual alignment (MA) calibration, where each sensor is accurately positioned on the body segment to assume a perfect match between the sensor reference frame and the bone-embedded reference frame (Bouvier et al., 2015; Höglund et al., 2021). The NP calibration is the most commonly adopted technique when working with IMU due to its simplicity and quickness in accomplishing a full-body calibration; for this reason, it can be found in most commercial motion capture systems (Choo et al., 2022; Roetenberg et al., 2013; Schepers et al.). FC calibration is more commonly found in research rather than commercial products, often due to the increased complexity and time required for the user to complete a full-body calibration. However, some studies have shown better joint angle accuracy for the elbow joint compared to other types of calibration (Cutti et al., 2008; Ligorio et al., 2017). Finally, MA calibration is less common in both research and commercial products; however, Bouvier and colleagues (Bouvier et al., 2015) found a similar accuracy performance of MA compared to other calibrations.

Each of these techniques has been presented and validated individually against reference systems. However, due to the complexity of the elbow joint, as well as numerous variables affecting measurement accuracy such as skin artefacts (Cappello et al., 2005; Cutti et al., 2005; Prabakaran and Rufus, 2022), misalignment between externally observed and anatomical reference frames (de Vries et al., 2010; Höglund et al., 2021) and sensor drift (LaViola, 2003), defining the best type of calibration to adopt for every real-life scenario remains challenging. Additionally, when measuring with IMU, the type of movement performed and the anatomical joint axis considered further exacerbate differences in joint angle estimation across calibrations. Therefore, this paper aims to compare the most commonly used calibration methods against an optical motion capture reference system during single-plane movements as well as multi-plane multi-joint movements to provide recommendations on the most suitable calibration method for each scenario.

## 2 Materials and methods

### 2.1 Subject recruitment

Thirteen healthy participants (age 27.6 ± 6.1, weight 64.0 ± 13.3 Kg, height 171.2 ± 6.1 cm), with no sign or pain or musculoskeletal injuries, were recruited at University Medical Centre Groningen. This study received approval from the ethical board of the University Medical Center Groningen, Groningen, Netherlands (nr RR10982) and was performed following the Declaration of Helsinki.

### 2.2 Subject instrumentation

**FIGURE 2:**
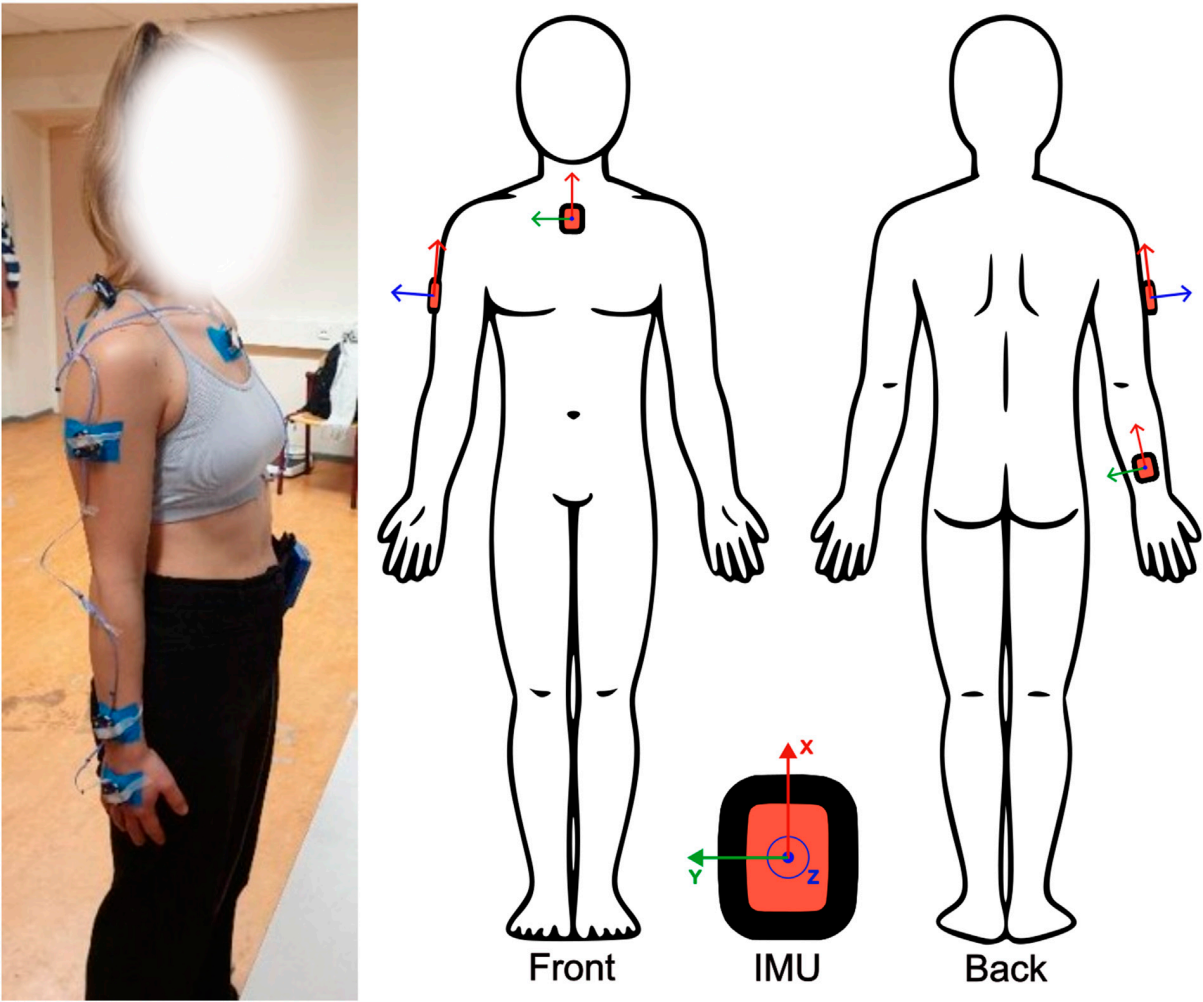
Sensor placement. IMU were placed on the sternum, upper arm, and forearm with reference axes aligned as shown. Active marker clusters were placed on top of each IMU on the same locations and secured with tape.

Each participant was instrumented with five IMU sensors (Movella DOT, Movella, Netherlands) on the sternum, scapula, upper arm, lower arm and hand on the right side of their body. The sternum IMU was placed below the incisura jugularis; The upper arm IMU was placed approximately at half the length of the humerus and facing laterally between the biceps and triceps muscle; The lower arm IMU was placed slightly above the wrist. These locations, as well as sensor reference axes are shown in Figure 2. Furthermore, one 3-marker optical marker cluster was placed on top of each IMU, which is connected to a 12-camera active optical motion capture system (Optotrak Certus^®^, NDI, Canada). Each pair of IMU and optical cluster were firmly secured on the participant’s skin using Kinesio Tape, while maintaining line-of-sight visibility between the active markers and the cameras (Figure 2).

### 2.3 System setup

#### 2.3.1 Optotrak

The optical motion capture system requires a landmark digitisation phase prior to data recording. This procedure consists of recording the position of a set of bony landmarks during a static pose, using a 4-marker rigid probe, in order to establish a relationship between the active marker cluster and each bony landmark. This method is further described in (van Andel et al., 2008), whereas the subsequent procedure to compute the elbow joint angles is in accordance with the International Society of Biomechanics (ISB) recommendations (Wu et al., 2005).

#### 2.3.2 IMU

Three different recordings were performed in order to acquire the IMU data necessary to perform the three different calibrations:

1) Static N-Pose: The subject stands upright and still for about 3 seconds whilst keeping: a) chest straight; b) arms completely straight and kept alongside the body with the palms touching the hips; c) feet parallel and about 20 cm apart.
2) Elbow flexion (de Vries et al., 2010): The subject is seated whilst keeping their elbow on a table. The olecranon is supported while the subject holds a long rigid stick with their hands at shoulder width and thumbs pointing laterally. The subject performs five elbow flexion/extension movements from about 15 to about 50 degrees of elbow flexion.
3) Elbow pronation (de Vries et al., 2010): The subject is seated with the olecranon supported on an armrest while their hand is free to move. The subject performs five elbow pronation/supination movements at full ROM without moving the olecranon from its fixed support.

### 2.4 Experimental protocol

Each subject performed five repetitions for each of the movements presented below. This procedure includes single-plane tasks and multi-joint tasks performed in the following order. The first four tasks in the list below were performed while the participants were standing.

1) Elbow flexion/extension: Starting from an N-pose, the subject performed elbow flexion/extension movements at full ROM with no constraints whilst limiting pronation movements as much as possible.
2) Elbow pronation/supination: Starting with the elbow flexed at 90° and the elbow touching the side of the body, the subject performed elbow pronation/supination movements at full ROM with no constraints whilst limiting flexion movements as much as possible.
3) Drinking: A paper cup of water was placed on a shelf at about eye height (1.6 m). Starting from an N-pose, the participant reached with their right arm towards the cup, grabbed it, brought it to their mouth, simulated drinking a sip of water and put the cup back on the shelf.
4) Box off-shelf: Starting from an N-pose, the participant stood in front of a shelf and moved a shoe box from a higher shelf (1.48 m height) to a lower shelf (0.96 m height) and then put it back.
5) Circles: The participant was seated and required to draw imaginary circles anticlockwise with their right arm by sliding a pen on a table in front of them. The participant was instructed to involve some degrees of shoulder, elbow and wrist motion without specifying the size of the circles.

### 2.5 Calibration reference frames

Three different initial reference frames are developed for the upper arm and lower arm segments associated with the NP, FC and MA calibration. Details of each computation are presented in Table 1.

• N-Pose (NP): The trunk sensor and the gravity vector serve as a reference to align each sensor. The advantage of this method is that the final segment reference frames solely depend on the trunk sensor’s orientation.
• Functional Calibration (FC): The joint rotation axes associated with elbow flexion and pronation are obtained from their relative gyroscope calibration data (Stančin and Tomažič, 2011). These rotation axes are then used to build the final segment reference frames for the upper arm and lower arm through cross-product multiplication, as described in Table 1.
• Manual Alignment (MA): Each sensor is carefully positioned on the body to ensure that the trunk and upper arm anatomical references are manually aligned with their respective sensor’s reference frame. These references are then rotated to match the ISB conventions as closely as possible (Table 1).

**TABLE 1:** Details of the joint axis calculations required to obtain the calibration quaternion $q_{0}$ from rotation matrix conversion for each calibration. Superscripts represent the reference system. G = global; I = inertial; UA = upper arm; LA = lower arm; a = acceleration; F/E = flexion/extension; P/S = pronation/supination. The orientation of the sensor local axis orientation is shown in Figure 2.

Segment	Primary axis	Secondary axis	Final axis definition
N-Pose			
Thorax	$G_{{TH}_{y}}=\left[0,0,1\right]$ : cranial	$S_{2}=G_{{TH}_{1}}\left[z\right]$ : forward	${\;}^{G}{TH}_{y}=\frac{S_{2}\times TH_{y}}{\left|S_{2}\times TH_{y}\right|}$ : lateral
			${\;}^{G}{TH}_{x}=\frac{TH_{y}\times TH_{z}}{\left|TH_{y}\times TH_{z}\right|}$ : lateral
Upper Arm			${\;}^{G}{TH}_{y}={\left({\;}^{G}R_{TH}\right)}_{\;Cali}$
Lower Arm			${\;}^{G}{TH}_{y}={\left({\;}^{G}R_{TH}\right)}_{\;Cali}$
Functional (Cutti et al., 2008)			
Upper Arm	${UA}_{z}={UA}_{{LA}_{F/E}}$ : lateral^a^	S2=UAaFEUAaFE : cranial	$UA_{x}=\frac{S_{2}\times UA_{z}}{\left|S_{2}\times UA_{z}\right|}$ : forward
			$UA_{y}=\frac{UA_{z}\times UA_{x}}{\left|UA_{z}\times UA_{x}\right|}$ : cranial
Lower Arm^b^	$LA_{y}=LA_{\;P/S}$ : forward	$S_{2}=\left[0,0,1\right]$ : cranial	$LA_{x}=\frac{LA_{y}\times S_{2}}{\left|LA_{y}\times S_{2}\right|}$ : forward
			$LA_{z}=\frac{LA_{y}\times S_{2}}{\left|LA_{y}\times S_{2}\right|}$ : lateral
Manual			
Upper Arm			${\;}^{G}{UA}_{x}={\;-}^{G}{UA}_{\;I}\left[y\right]$ :forward
			${\;}^{G}{UA}_{y}={\;}^{G}{UA}_{1}\left[x\right]$ :cranial
			${\;}^{G}{UA}_{z}={\;}^{G}{UA}_{1}\left[z\right]$ : lateral
Lower Arm			${\;}^{G}{LA}_{x}={\;-}^{G}{LA}_{\;I}\left[y\right]$ :forward
			${\;}^{G}{LA}_{y}={\;}^{G}{LA}_{1}\left[x\right]$ :cranial
			${\;}^{G}{UA}_{z}={\;}^{G}{LA}_{1}\left[z\right]$ : lateral

^a^ Refers to the joint rotation axis computed with the lower arm sensor and translated into the upper arm reference.

^b^ The direction of the rotation axis refers to the body placed in an upright position and the elbow flexed at about 90°.

### 2.6 Data analysis

**FIGURE 3:**
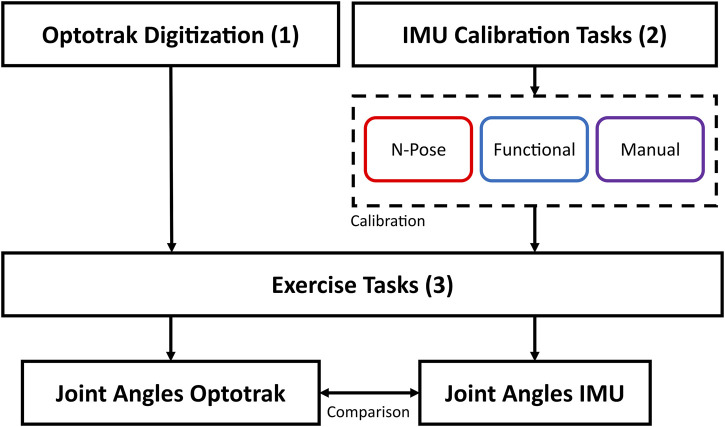
Graphical representation of data collection and analysis. The OPTO system was calibrated through bony landmark digitisation. IMU data was calibrated through 1) N-Pose calibration, 2) Functional Calibration, 3) Manual alignment. Subsequently, joint angle data was collected with both systems and finally compared.

Data analysis was performed in MATLAB (The MathWorks Inc, Massachusetts, US; version R2022b). The Optotrak elbow joint angle was calculated following the ISB recommendations by choosing the humerus H2 model for the elbow pronation task and H1 for all other tasks (Wu et al., 2005). The glenohumeral joint rotation centre necessary to define the humerus was computed using (Rab et al., 2002) because it was shown to be the most accurate (Michaud et al., 2016). Considering the IMU data, each calibration was processed individually to compute a calibration quaternion $q_{0}$ that is then multiplied by the runtime sensor data (Eq. 1) to produce the real-time elbow joint angle. The data analysis workflow is also presented in Figure 3. We then computed the Range of Motion (ROM) error, Root Mean Squared Error (RMSE) and offset by comparing optical (reference) and IMU joint angle data. ROM error was calculated as the difference between the Optical and IMU ROM (Eq. (2)). RMSE was calculated as shown in Equation (3), where $\hat{\theta }$ is the mean joint angle within each repetition. Offset is the difference between optical and IMU mean joint angle within the repetition (Eq. (4)).

$${GS}_{q}={GI}_{q_{runtime}}⊗\left({\;}^{SI}q_{0}\right)$$ (1)

$$ROM\;error={\left(\theta_{\mathrm{Max}}-\theta_{\mathrm{Min}}\right)}_{OPTO}\;-{\left(\theta_{\mathrm{Max}}-\theta_{\mathrm{Min}}\right)}_{IMU}$$ (2)

$$RMSE=\sqrt{\frac{\sum_{i=1}^{N}{\left|\left(\theta_{OPTO}\left(i\right)\;-\;{\hat{\theta }}_{OPTO}\right)\;-\;\left(\theta_{IMU}\left(i\right)\;-\;{\hat{\theta }}_{IMU}\right)\;\right|}^{2}}{N}}$$ (3)

$$Offset={\hat{\theta }}_{OPTO}\;-{\hat{\theta }}_{IMU}$$ (4)

### 2.7 Statistical analysis

A three-way repeated-measures ANOVA was performed to evaluate the effect of joint angle axis and movement tasks on the three calibrations. We performed three separate analyses for each dependent variable (ROM error, RMSE and offset) and chose the following within-subject factors: 1) Calibrations (NP, FC, MA); 2) Axes (flexion, pronation, carrying angle); 3) Tasks (flexion, pronation, drinking, box-off-shelf, circles). The significance level was set at α=0.05 , and the Generalised eta squared ( $\eta_{\;G}^{2}$ ) was chosen to calculate the effect size (Bakeman, 2005), which is interpreted as η^2^_G_ = 0.02 as a small effect, η^2^_G_ = 0.13 as a medium effect and η^2^_G_ = 0.26 as a large effect (Cohen, 1988). We applied Greenhouse-Geisser to correct the degrees of freedom whenever the sphericity assumption was violated. The statistical analysis was performed in JASP (JASP Team 2023; version 0.17.2.1).

## 3 Results

**FIGURE 4:**
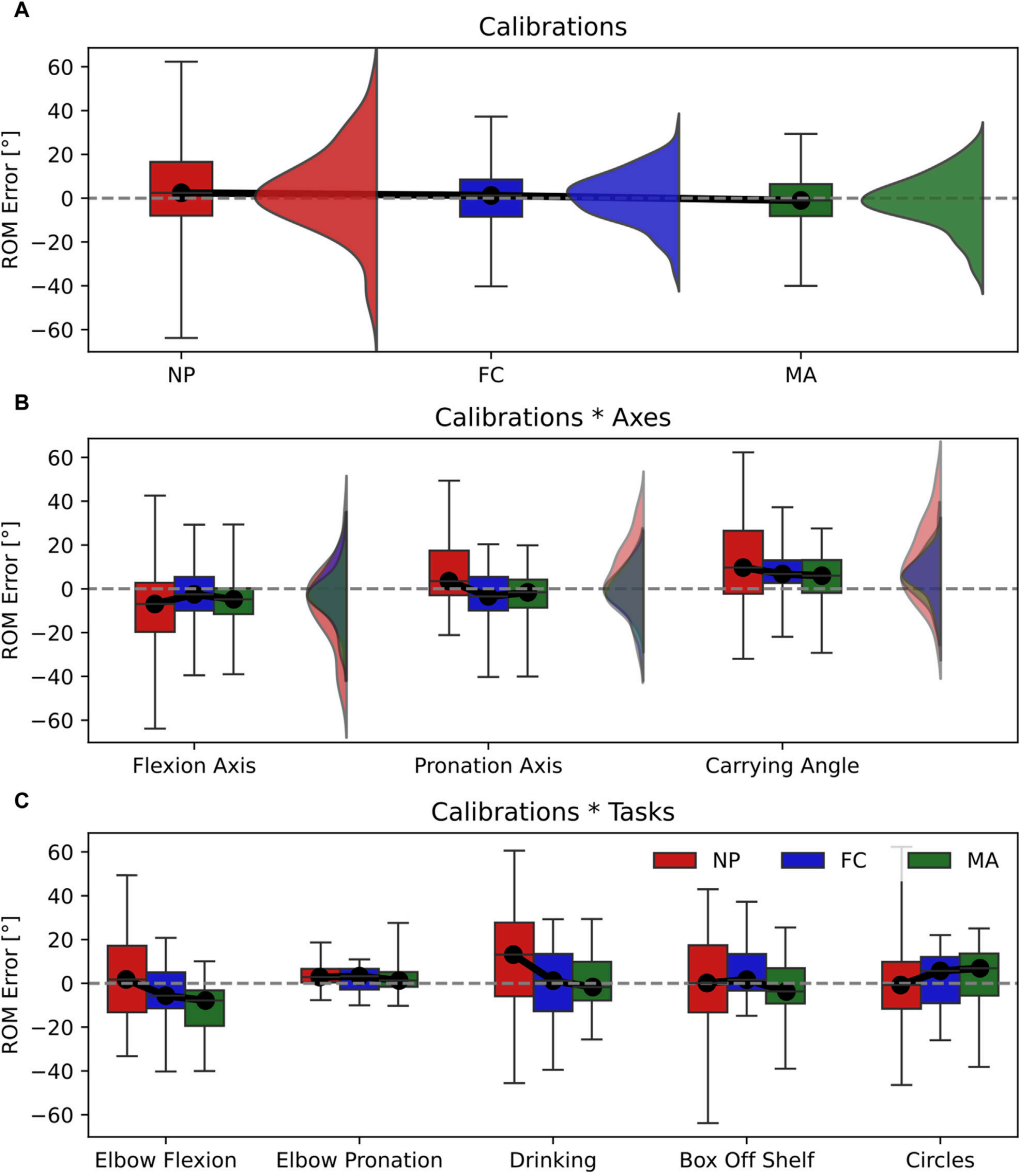
Box-and-whisker plots representing the ROM error for the three calibrations (NP, FC, MA). **(A)** Main effect of calibrations; **(B)** interaction effect Calibrations*Axes; **(C)** interaction effect Calibration*Tasks. Graphs A and B include violin plots to show data distribution.

The ROM error computed with the three calibrations displayed significant differences in the main effect and interaction effects for both axes and tasks (Table 2). However, their effect size is small, thereby, indicating a small influence of different calibration techniques on the overall ROM error. Interestingly, while the accuracy between calibration is comparable, NP was the least precise calibration over participants. This can be observed by a larger interquartile range of NP (IQR = 24.5°) compared to FC (IQR = 16.98°) and MA (IQR = 14.59°) in the main effect (Figure 4A). A similar trend can be observed in the calibration*axes interaction (Figure 4B) on the flexion axis (NP IQR = 22.42°) and carrying angle (NP IQR = 28.74°) and in the calibration*tasks interaction (Figure 4C) during elbow flexion (NP IQR = 30.38°), drinking (NP IQR = 33.63°) and box-off-shelf (NP IQR = 30.62°). However, the effect sizes of all these effects are rather small and should not be overinterpreted.

**TABLE 2:** Results of the three-way repeated-measures ANOVAs for ROM error, RMSE, offset, displaying degrees of freedom (df), F-ration (F), *p*-value (p) and generalised eta squared (η^2^_G_). Asterisks indicate statistically significant differences (*p* < .05).

Cases	df	F	p	η^2^_G_
ROM Error				
Calibrations	1.819	6.043	0.004*	0.025
Calibrations*Axes	3.637	6.242	<0.001*	0.051
Calibrations*Tasks	7.274	2.978	0.005*	0.049
RMSE				
Calibrations	1.632	28.644	<0.001*	0.125
Calibrations*Axes	3.264	1.773	0.148	0.017
Calibrations*Tasks	6.529	85.926	<0.001*	0.082
Offset				
Calibrations	1.881	52.670	<0.001*	0.136
Calibrations*Axes	3.763	43.964	<0.001*	0.208
Calibrations*Tasks	7.526	2.834	0.006*	0.033

**FIGURE 5:**
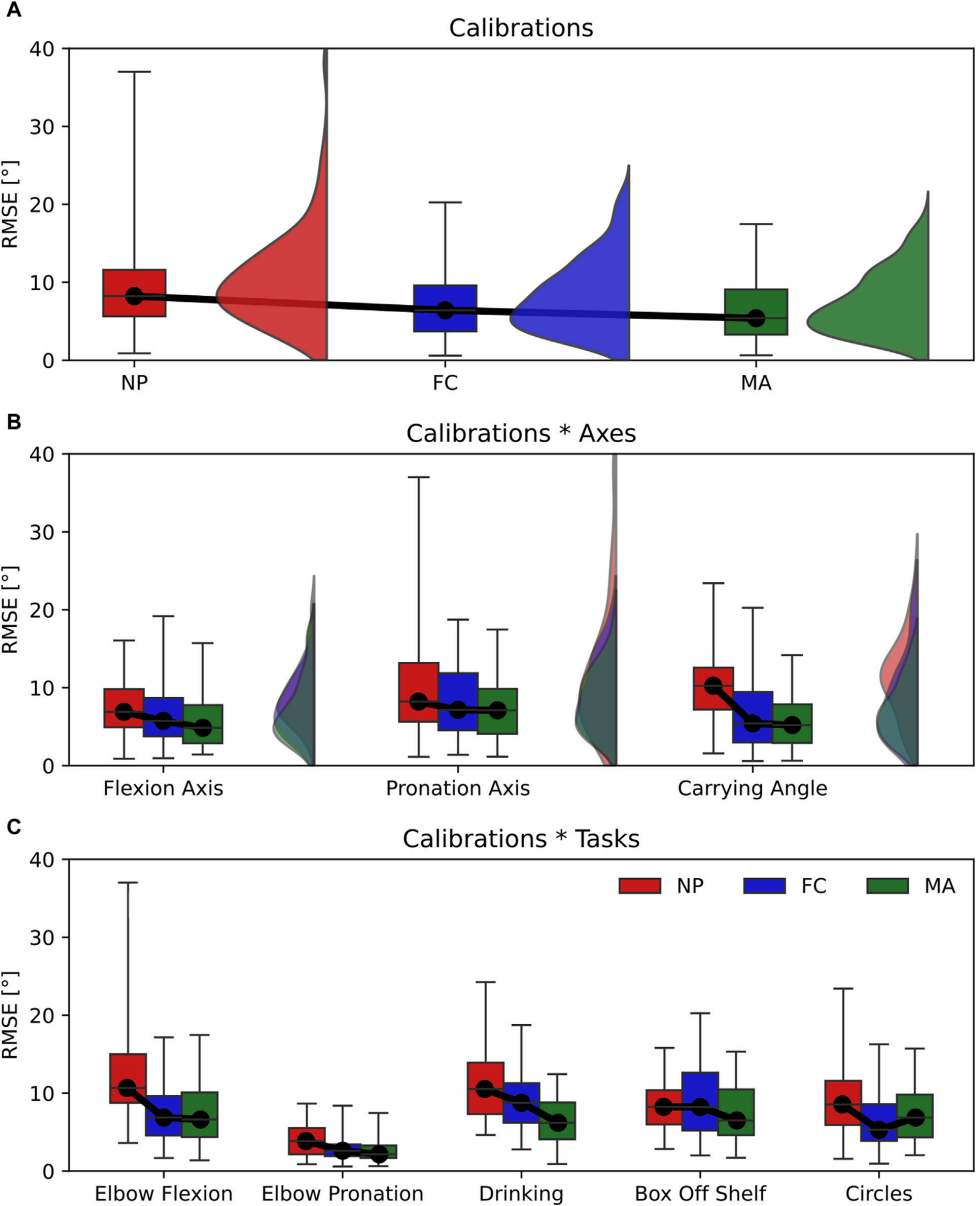
Box-and-whisker plots representing the RMSE for the three calibrations (NP, FC, MA). **(A)** Main effect of calibrations; **(B)** interaction effect Calibrations*Axes; **(C)** interaction effect Calibration*Tasks. Graphs A and B include violin plots to show data distribution.

The RMSE displayed significant differences between calibrations in the main effect with a relatively large effect size (Table 2). This is shown in Figure 5A where the RMSE computed for NP (RMSE = 8.2°) is significantly larger than FC (RMSE = 7.2°) and MA (RMSE = 6.3°). In addition, differences between calibrations are observed in the calibrations*tasks interaction (Figure 5C), which also yielded a relatively large effect size. Specifically, NP displayed larger RMSE values in elbow flexion, elbow pronation, drinking and circles tasks compared to FC and MA. On the other hand, MA appears superior to FC as it showed a lower RMSE, by approximately 1°, compared to FC in the main effect, as well as calibrations*tasks interaction during elbow pronation, drinking and box-off-shelf.

**FIGURE 6:**
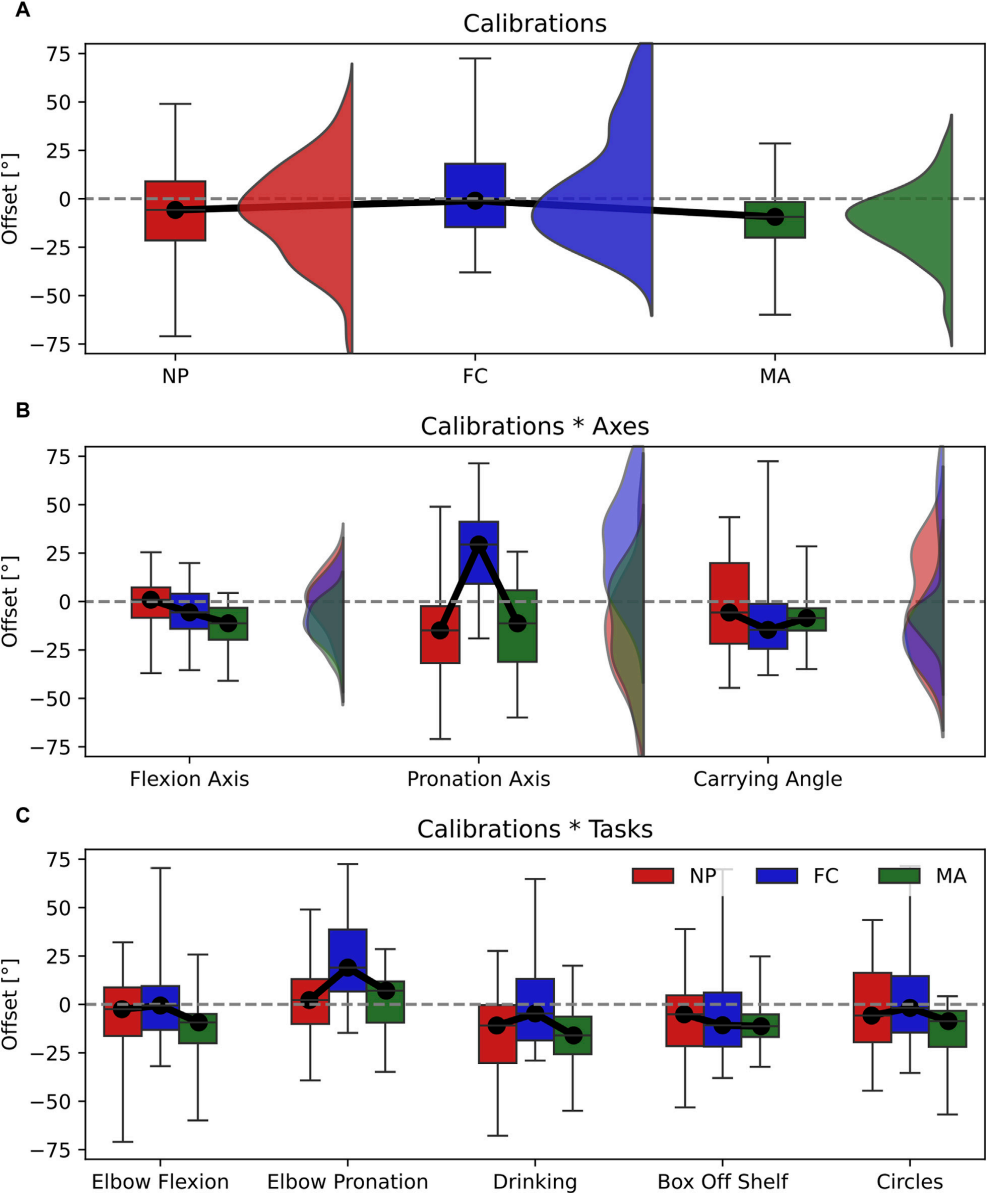
Box-and-whisker plots representing the Offset for the three calibrations (NP, FC, MA). **(A)** Main effect of calibrations; **(B)** interaction effect Calibrations*Axes; **(C)** interaction effect Calibration*Tasks. Graphs A and B include violin plots to show data distribution.

Joint angle offset showed significant differences between the three calibrations in the main effect with a relatively large effect size (Table 2). This can be observed in Figure 6A as FC is the most accurate calibration (Offset = −1.0°), followed by NP (Offset = −5.8°) and MA (Offset = −9.4°). In this context, the joint angle axis has a significant influence on the overall offset as the calibrations*axes interaction effect yielded a relatively large effect size (Figure 6B). Specifically, NP is the most accurate calibration on the elbow flexion axis (offset = 1.0°) and carrying angle (offset = −5.6°) and is comparable to MA whereas FC performed the worst on the same axes respectively by approximately 5°. Overall MA appeared as the most consistent and reliable calibration across different joint axes.

## 4 Discussion

In this work, we have highlighted the influence of sensor-to-segment calibration on joint angle estimation with IMU sensors. In particular, we explored the impact of calibration in two main conditions: 1) joint angle accuracy across different anatomical planes, or joint axes, namely, flexion axis, pronation axis and carrying angle; 2) type of task performed, namely, pure elbow flexion, pure pronation and multi-joint tasks that include different combinations of flexion and pronation. Choosing an appropriate calibration method is not trivial since our results show that calibration performance can vary broadly depending on the 3D joint angle axis considered or the movement performed. For instance, the same type of calibration can produce different RMSE values as large as 10° when changing the movement performed from elbow flexion to pronation (RMSE NP elbow flexion = 13.37° ± 8.02°; RMSE NP elbow pronation = 3.98° ± 2.12°, see Figure 5C). Conversely, varying the joint angle axis has a lower impact on RMSE as differences are within 4° when using the same type of calibration (i.e., RMSE NP flexion axis = 7.47° ± 3.42°; RMSE NP pronation axis = 10.31° ± 7.94°, see Figure 5B).

Our results showed that each type of calibration performs uniquely depending on the variable, joint axis and movement tasks considered. For ROM estimation, varying the type of calibration in all tasks and joint angle axes resulted in small effect sizes, thereby only minor differences in performance (Figure 4A). RMSE showed non-significant differences in calibration*axes interaction effect, indicating no difference in performance on all joint angle axes (Figure 5B). However, varying the task performed resulted in significant differences among calibrations and a moderate effect size. In particular, NP displayed an RMSE larger than 5° compared to FC and MA when the elbow moves in pure flexion or in multi-joint tasks (Figure 5C). In these latter the flexion component is dominant, thereby a large RMSE for NP is expected. For these tasks FC and MA are valid alternatives as both display lower mean RMSE values and smaller interquartile ranges. Offset showed significant differences and a large effect size in the calibration*axes interaction (Figure 6B), indicating substantial variations in the performance of the calibrations on different joint angle axes. In particular, FC calibration appears less reliable than the other calibrations on pronation movements and joint pronation axis when estimating joint angles. Still, it is the most accurate on flexion movements and multi-plane tasks.

The ROM error, RMSE and offset variables analysed all play an essential role in the clinical rehabilitation field, as clinicians aim for the most accurate measurements with standardised assessment movements (single plane tasks such as pure elbow flexion and pronation) as well as measurements in real-life conditions, such as multi-joint tasks. Therefore, providing recommendations on the best IMU calibration technique is imperative to drive accurate clinical diagnoses on musculoskeletal movement conditions (Bo et al., 2022; Zadeh et al., 2023). We provide our final considerations for each calibration technique analysed in this paper in the following paragraph:

• **NP** is generally the most common type of calibration for commercial motion capture products (Roetenberg et al., 2013) as well as clinical rehabilitation products (Choo et al., 2022). Considering our results on the NP calibration, we often observe the highest errors and standard deviations on joint angle estimation across a wide range of joint axes and movement tasks. Therefore, we advise against using NP for the most accurate estimation of elbow joint angles.
• **FC** relies on the execution of strict single-plane elbow flexion and pronation movements for IMU calibration. This procedure can be an optimal solution for patients who are unable to maintain a fixed posture, such as neurological disorders (Hsu et al., 2018) or severe postural abnormalities (Petropoulos et al., 2020; Tisler and Kozlovszky, 2022), since only small movements can suffice to achieve a correct IMU calibration. Data shows that FC is most accurate on pure elbow flexion tasks as well as multi-plane tasks that have a predominant flexion component, whereas it produces a large offset on pronation. We advise the use of FC when the focus of the measurement is on achieving the most accurate elbow flexion angle.
• **MA** requires accurate sensor positioning and alignment of the sensors on the wearer’s body segments (Fang et al., 2023), requiring extensive training of the operator. MA displays good accuracy and low errors across a multitude of joint angle axes and during both single-plane and multi-plane tasks. Therefore, we recommend MA as the preferred elbow joint calibration method for general use in rehabilitation.

### 4.1 Relevance of carrying angle in elbow modelling

Most of the literature on elbow biomechanical modelling with IMU represents the elbow as a double-hinge joint (Cutti et al., 2006; Cutti et al., 2008; Ligorio et al., 2017), which allows for elbow flexion/extension and pronation/supination movements and, thereby, neglecting the carrying angle (Figure 1). However, it is known that the carrying angle can vary depending on the age, sex and anatomy of the individual, and it is a function of the elbow flexion angle that can exhibit linear or sinusoidal patterns (An et al., 1983). Therefore, ignoring its influence on the flexion and pronation axes can introduce non-negligible crosstalk errors, as demonstrated by Piazza and colleagues (Piazza and Cavanagh, 2000). Furthermore, the study of the carrying angle can find applications in several fields, including prosthetics development (Stokdijk et al., 1999).

In this work, we model the elbow as a three-degree-of-freedom joint, including the computation of the carrying angle. We report metrics about this specific joint axis for completeness to the reader and highlight one of its potential applications in IMU modelling: rating the accuracy of the IMU calibration. Since the carrying angle can vary by up to 15° when the elbow ranges from full extension to full flexion (An et al., 1983), detecting its deviations beyond acceptable ranges is crucial to identify crosstalk errors caused by a non-optimal calibration. Consequently, it can help mitigate the risk of collecting unusable patient data in a clinical setting.

## 5 Conclusion

Estimating elbow joint angles using IMU presents unique challenges as varying movement tasks, and joint angle axes can largely influence the accuracy of the measurement. This study compares three sensor-to-segment calibration methods to guide the user in choosing the most appropriate calibration type depending on their goal. Whilst the performance of each calibration is similar for ROM measurements, they widely differ in RMSE and offset. In particular, NP calibration often yields the highest RMSE errors, whereas FC and MA show the lowest errors across many joint angle axes and movement tasks. Therefore, we advise using MA as the preferred calibration method for the elbow joint, which relies on accurately placing the sensors on the wearer’s upper and lower arm. Alternatively, FC proves advantageous when the wearer cannot hold a known posture (i.e., in patients with severe postural abnormalities) because it relies on the execution of elbow flexion and pronation movements for calibration.

## Data Availability

The full IMU and optical motion capture dataset can be downloaded here: https://doi.org/10.5281/zenodo.10935873. DOI: 10.5281/zenodo.10935873. This also includes a more detailed explanation of the experimental protocol as well as MATLAB example codes.
